# The *wzc* mutation mediates virulence changes in K1-type *Klebsiella pneumoniae* within the same patient

**DOI:** 10.3389/fmicb.2025.1577629

**Published:** 2025-05-15

**Authors:** Gaoqin Teng, Qiuying Qin, Shuo Ding, Yanchao Wu, Yingying Fu, Meng Zhang, Xiaoqiang Yang, Ye Jin, Zhijiang Xu, Man Huang

**Affiliations:** ^1^Department of General Intensive Care Unit, The Second Affiliated Hospital of Zhejiang University School of Medicine, Hangzhou, China; ^2^Key Laboratory of Multiple Organ Failure (Zhejiang University), Ministry of Education, Zhejiang, China; ^3^Department of Immunology and Pathogen Biology, School of Basic Medical Sciences, Hangzhou Normal University, Hangzhou, China

**Keywords:** hypervirulent *Klebsiella pneumoniae*, virulence, *wzc*, capsular polysaccharide, mucoid

## Abstract

Hypervirulent *Klebsiella pneumoniae* (hvKp) is a major pathogen causing community-acquired infections, particularly severe diseases such as liver abscesses. Although extensive research has been conducted on the virulence mechanisms of hvKp and the genetic properties of resistance plasmids, studies on the adaptive evolution of clinical strains within the host are still limited. This study aimed to investigate the impact of genetic mutations on phenotypic changes in high-virulence *K. pneumoniae* within a host environment. We isolated three strains of *K. pneumoniae* from the same patient, two of which had identical genetic backgrounds but exhibited distinct phenotypic traits. Comparative genomic analysis was performed to identify genetic differences. A nucleotide mutation in the wzc gene was identified as a potential factor associated with changes in the mucoid phenotype. This mutation was verified using string tests and anti-centrifugal assays. Additionally, *in vivo* bioassays and animal infection models were conducted to further validate the findings. The comparative genomic analysis revealed a nucleotide mutation in the wzc gene, which was associated with changes in the mucoid phenotype of the strain. This was confirmed through string tests and anti-centrifugal assays. *In vivo* experiments and animal infection models suggested that hvKp adapts to the host by reducing capsular polysaccharide synthesis, thereby trading off some virulence for enhanced colonization capabilities. Our findings indicate that genetic mutations in hvKp can lead to significant phenotypic changes that facilitate adaptation within the host. The observed reduction in capsular polysaccharide synthesis appears to be a trade-off between virulence and colonization ability. This study provides insights into the adaptive evolution of hvKp and highlights the importance of considering intrahost genetic changes when studying the pathogenesis of hvKp. Future research should focus on further elucidating the mechanisms underlying these adaptations and their clinical implications.

## Introduction

*Klebsiella pneumoniae* (*K. pneumoniae*) is an opportunistic clinical pathogen responsible for a wide range of infections, particularly in patients with compromised immune systems (Paczosa and Mecsas, 2016). Based on virulence, *K. pneumoniae* is broadly classified into two categories: classic *K. pneumoniae* (cKp) and hypervirulent *Klebsiella pneumoniae* (hvKp). hvKp infects healthy individuals of all ages and has the potential to disseminate to multiple body sites, leading to severe invasive infections, including pneumonia, renal abscesses, liver abscesses, brain abscesses, and bloodstream infections (Yang et al., 2020; Dong et al., 2022; Gu et al., 2018). *K. pneumoniae* possesses a repertoire of virulence-associated factors that significantly contribute to its pathogenicity. These include important virulence factors, such as capsular polysaccharides (CPS), which enclose a protective cover over the outer membrane of bacteria and resist attack by the host’s immune system (Liu et al., 2025); siderophores, which capture iron—a critical necessity for bacterial survival and development (Namikawa et al., 2022; Russo et al., 2021); fimbriae, which facilitate adhesion to cells in the host (Tong et al., 2024; Lavender et al., 2005); lipopolysaccharides (LPS), which contribute to endotoxin production and immune attack evasion (Hwang et al., 2025); and inner membrane transport proteins, which aid in the absorption of essential nutrients necessary for bacterial survival and proliferation within the host environment (Chen et al., 2023). Genetic diversity and capsular polysaccharide expression can arise in numerous capsular serotypes, known as K antigens. To date, at least 160 distinct K antigens have been identified (Lam et al., 2022). The primary hypervirulent *K.* capsular types include K1, K2, K20, K54, and K57 (Russo et al., 2018; Wei et al., 2021). Its use in intensive care unit (ICU) environments is widespread, and it is a cause of considerable public concern for health (Lan et al., 2021; Liu et al., 2022; Lee et al., 2016). ICU management can become increasingly complicated with the rise of multidrug-resistant (MDR) strains of *Klebsiella pneumoniae* that have enhanced virulence factors, along with an increase in MDR strains and hvKP. Sources of *Klebsiella pneumoniae* infection may include central venous catheterization, mechanical ventilation, tracheotomy, or the use of urinary catheters.

Main virulence factors of hvKp include *rmpACD, rmpA2, aerobactin (iucABCD, iutA), salmochelin (iroBCDNE), rmpC, rmpD,* and *peg344* (Carlos Catalan-Najera et al., 2017; Lopez et al., 2020). Among these, *rmpACD* and *rmpA2* play a key role in regulating CPS expression and mucoidy, conferring a hypermucoviscous phenotype to the organism (Choby et al., 2020). The increase in mucoid viscosity can also be observed in the absence of *rmpA1* and *rmpA2* and with different capsular serotypes, indicating that there are other genetic determinants of mucoid viscosity in *Klebsiella pneumoniae* (Beckman et al., 2024). In addition, infection-related genes, including siderophore-related genes such as aerobactin and salmochelin, as well as virulence-promoting genes, such as *rmpC, rmpD,* and *peg344,* play important roles in infection and virulence, respectively (Choby et al., 2020). Recent studies have revealed that mutations in capsular synthesis genes and polymorphisms in nucleic acid in the *rmpA* promoter region influence capsular synthesis and, consequently, strain virulence (Ernst et al., 2020).

The high-mucoid phenotype is a key feature of hypervirulence (Lai et al., 2003). CPS can protect bacteria from the attacks of the host’s immune system, particularly by resisting the complement system and avoiding recognition and clearance by phagocytic cells. The synthesis of CPS in *K. pneumoniae* is encoded by genes located on chromosomal operons. The CPS gene cluster contains several genes, including *wzi*, *wza*, *wzb*, *wzc*, *gnd*, *wca*, *cpsB*, *cpsG*, and *galF*, all of which are involved in CPS synthesis (Pan et al., 2008). The *wzi*, *wza*, and *wzc* genes are involved in the transport and assembly of CPS. The *wzi* gene is responsible for transferring CPS from the cytoplasmic side to the periplasmic side, *wza* forms an outer membrane channel, and *wzc* acts as a tyrosine kinase that participates in the synthesis and regulation of CPS chains (Whitfield, 2006; Whitfield et al., 2020). The enzyme encoded by the *gnd* gene is involved in the synthesis of CPS precursors (Matsuoka and Shimizu, 2012). The *wca* gene cluster, particularly *wcaJ*, is directly involved in CPS synthesis, while the *cpsB and cpsG* genes may play a role in the processing. The product of the *galF* gene functions in the biosynthesis of the carbohydrate components of CPS (Peng et al., 2018). These genes and their protein products collaborate to ensure the correct synthesis and function of CPS, playing a crucial role in the virulence, immune evasion, and host persistence of the bacteria. Intriguingly, although hvKp can cause more severe damage clinically, the *K. pneumoniae* strains isolated in clinical settings primarily exhibit a hypermucoviscous phenotype. Currently, there is insufficient evidence to elucidate the relationship between changes in the mucoviscous phenotype of *K. pneumoniae* and its adaptability for colonization and survival within the host.

In this study, we cultivated three distinct strains of *Klebsiella pneumoniae* from a clinical sample obtained from a patient in the general intensive care unit (GICU). Using filamentation assays and centrifugation tests, we observed significant phenotypic variations among these strains. Further analyses using whole genome sequencing (WGS), gene knockout techniques, and animal infection models revealed the critical regulatory role of *wzc* gene polymorphisms in modulating bacterial virulence and mucoidy. This genetic diversity may confer enhanced adaptability to the host environment. Our findings highlight the pivotal role of *wzc* polymorphisms in the evolutionary adaptation of *K. pneumoniae* within the host and suggest new avenues for clinical surveillance and therapeutic strategies against hvKP.

## Materials and methods

### Bacterial strains and growth conditions

This study utilized three *Klebsiella pneumoniae* strains isolated from the sputum of a GICU-hospitalized patient (Table 1), and 16S rDNA sequencing was conducted to verify the identity of the *Klebsiella* isolates. The strains were stored at −80°C in 50% (v/v) glycerol, and all cultures were amplified at 37°C with shaking at 200 rpm in lysogeny broth (LB) culture medium.

**Table 1:** Comparison of the antibiotic resistance profiles of the three strains of *Klebsiella pneumoniae.*

Antibiotics	GICU_KP01		GICU_KP02		GICU_KP03	
	MIC	Sensitivity	MIC	Sensitivity	MIC	Sensitivity
Tigecycline	≤0.5	S	≤0.5	S	2	S
Imipenem (IPM)	≤0.25	S	≤0.25	S	≥16	R
Meropenem (MEM)	≤0.25	S	≤0.25	S	≥16	R
Polymyxin	≤0.5	S	≤0.5	S	≤0.5	S
Piperacillin/Tazobactam (TZP)	≤4	S	≤4	S	≥128	R
Amikacin (AMK)	≤2	S	≤2	S	≥64	R
Tobramycin (TOB)	≤1	S	≤1	S	≥16	R
Cefoperazone/Sulbactam (SFP)	≤8	S	≤8	S	≥64	R
Aztreonam (ATM)	≤1	S	≤1	S	≥64	R
Ciprofloxacin (CIP)	≤0.25	S	≤0.25	S	≥4	R
Levofloxacin (LVX)	≤0.12	S	≤0.12	S	≥8	R
Ceftazidime (CAZ)	0.25	S	0.25	S	≥64	R
Minocycline (MNO)	4	S	4	S	≥16	R
Doxycycline	2	S	2	S	8	I
Cefepime (FEP)	≤0.12	S	≤0.12	S	≥32	R
Puromyn	≤20	S	≤20	S	≤20	S

### Antimicrobial susceptibility tests

Antimicrobial susceptibility testing and interpretation were performed according to the guidelines of the Clinical and Laboratory Standards Institute (CLSI). Carbapenem resistance was defined as resistance to ertapenem [minimum inhibitory concentration (MIC > 2 mg/mL) and resistance to imipenem or meropenem (MIC > 4 mg/mL)] (CLSI, 2021).

### String test

The string test was conducted following the method described by Yao et al. (2015). The three strains of *Klebsiella pneumoniae* were streaked onto Columbia blood agar plates and incubated overnight at 37°C. A positive result in the filamentation assay was defined by a viscous filament length exceeding 5 mm, while a negative result was defined by a filament length of less than 5 mm. Statistical comparisons were conducted using GraphPad Prism version 8.00, employing Student’s *t*-test to assess the differences.

### Growth rate determination

To compare the growth rates of the different bacterial strains, the monoclonal strains GICU_KP01, GICU_KP02, and GICU_KP03 were transferred to antibiotic-free LB broth and incubated at 37°C with shaking at 200 rpm (Pollack-Milgate et al., 2024). The specific steps were as follows: The overnight cultures of the bacteria were diluted 1:1,000 into fresh LB broth, and the optical density at 600 nm (OD600) was measured hourly using a spectrophotometer (Spectrum Lab S32A) for a total duration of 24 h.

### Pulsed-field gel electrophoresis

Molecular typing was performed using pulsed-field gel electrophoresis (PFGE), following the method described previously (Li et al., 2021). In brief, DNA from each bacterial strain was embedded in agarose blocks, with three to four mini-gels prepared for each bacterial sample, each containing a volume of approximately 100μL, and allowed to solidify at 4°C for 30 min. Following the PulseNet standards, *Salmonella enterica* serotype Braenderup strain H9812 was used as the PFGE molecular weight standard. The DNA blocks were digested with the restriction enzyme *XbaI* and electrophoresed at 14°C for 18 h in a 0.5 × TBE buffer using the CHEF Mapper XA system (Bio-Rad, United States). After staining with ethidium bromide, the DNA fragments were visualized using GelCompar II (Bio-Rad, United States).

### Biofilm formation

Monoclonal strains—GICU_KP01, GICU_KP02, and GICU_KP03—were picked and transferred to fresh LB medium, followed by incubation at 37°C with shaking at 220 rpm. The overnight cultures were diluted in fresh LB medium at a ratio of 1:1,000, and 200 μL of the diluted bacterial suspension was then added to each well of a 96-well polystyrene plate (Ashwath et al., 2022). After static incubation at 37°C for 48 h, each well containing the sample was washed with double distilled water (ddH_2_O) and the biofilm was fixed with 99% methanol. Then, 200 μL of a 0.1% crystal violet staining solution was added to the sample wells and stained for 10 min. The supernatant was gently aspirated and discarded using a syringe, followed by three washes with ddH_2_O. Finally, the crystal violet was dissolved in 200 μL of 95% ethanol, and the optical density at 570 nm was measured. Statistical comparisons were conducted using GraphPad Prism version 8.00, with Student’s *t*-test employed to assess the differences.

### Whole genome sequencing and bioinformatics analysis

As previously described, genomic DNA from the aforementioned strains was extracted and sequenced using the Illumina HiSeq platform (MEIGE, Guangzhou). Long-read libraries for GICU_KP01, GICU_KP02, and GICU_KP03 were created using the Nanopore platform (Oxford Nanopore Technologies, Oxford, UK). To achieve complete genome assemblies for GICU_KP01, GICU_KP02, and GICU_KP03, the HGAP workflow in SMRT Analysis v2.3.0 was employed to assemble long and short reads. The resulting genomic sequences were annotated using RAST v2.0.^1^ Plasmid incompatibility was investigated using PlasmidFinder v2.1,^2^ and resistance genes were identified using ResFinder v.4.1.^3^ Insertion sequences were predicted using ISfinder,^4^ and conjugative elements were identified using oriTfinder^5^ to assess the conjugative potential of the plasmids. Large overlapping sequences were identified through BLASTN searches (Overbeek et al., 2014). The BRIG program was used to visualize similar plasmid maps (Alikhan et al., 2011).

### Mouse infection model

The pathogenicity of the different *Klebsiella pneumoniae* strains was evaluated using a murine bacteremia model (Endimiani et al., 2011). Female CD-1 mice (average weight approximately 20 g, 6 weeks) were purchased from Hangzhou Qingzhen Laboratory Animal Technology Co. Ltd. (Hangzhou, China). Throughout the study, the mice were provided with specialized laboratory animal feed and ddH_2_O. The experiment consisted of four groups, each corresponding to a different *Klebsiella pneumoniae* strain, with eight mice per group. A murine bacteremia model was established via intraperitoneal injection to evaluate pathogenicity, with each mouse receiving 10^7^ CFU of *Klebsiella pneumoniae*. The mice were monitored for 180 h post-infection, and the mortality rate was recorded. Survival curves were generated using GraphPad Prism v.8.00. Statistical analyses were conducted using the log-rank test (Mantel-Cox), following the recommendations of Prism v.8.00.

### *wzc* knockout and complementation strains

Gene knockout in *Klebsiella pneumoniae* was performed using the CRISPR/Cas9 editing system (Teng et al., 2024). To knock out the *wzc* capsule polysaccharide synthesis gene in GICU_KP02, 450 bp of the upstream and downstream regions of the *wzc* gene on the GICU_KP02 genome were amplified to serve as donor DNA for homologous recombination fragments. The pSGKP plasmid extracted from *Escherichia coli* DH5α was then ligated with the *wzc* gene using a homologous recombination method. Subsequently, it was co-electroporated into GICU_KP02, which carried the pCasKP plasmid, along with the upstream and downstream homologous recombination fragments. The correct mutants were verified using PCR and Sanger sequencing. The *wzc* gene from the GICU_KP01 genome was amplified using PCR and ligated with the pSGKP plasmid through homologous recombination. Then, it was electroporated into the GICU_KP02 strain, in which the *wzc* gene had been knocked out. The plasmids and primers used are listed in Supplementary Tables S1, S2.

### Protein structure prediction and alignment

We used Alphafold3^6^ to predict the structure of the tyrosine protein kinase by entering the amino acid sequence of *wzc* on the website, initiating the task, and then awaiting the prediction results. The predicted model was imported into PyMOL for protein structure alignment.

## Results

### Patient hospitalization baseline information

In November 2018, a 61-year-old male individual was hospitalized via ambulance following a traffic accident that left him unconscious. Upon admission, he was diagnosed with a pulmonary contusion, severe traumatic brain injury, and a cervical spine fracture and was assigned a Glasgow Coma Scale (GCS) score of 1 + T + 3. He was intubated and placed on mechanical ventilation, then transferred to the general intensive care unit (GICU) for further management. During the GICU stay, he developed acute respiratory distress syndrome (ARDS) secondary to a pulmonary infection and received symptomatic treatment. On the 17th day, with stabilized vital signs, he was moved to the rehabilitation department. After assessing his response to rehabilitation exercises for a day, he was transferred to a specialized rehabilitation hospital for ongoing recovery.

**Figure 1:**
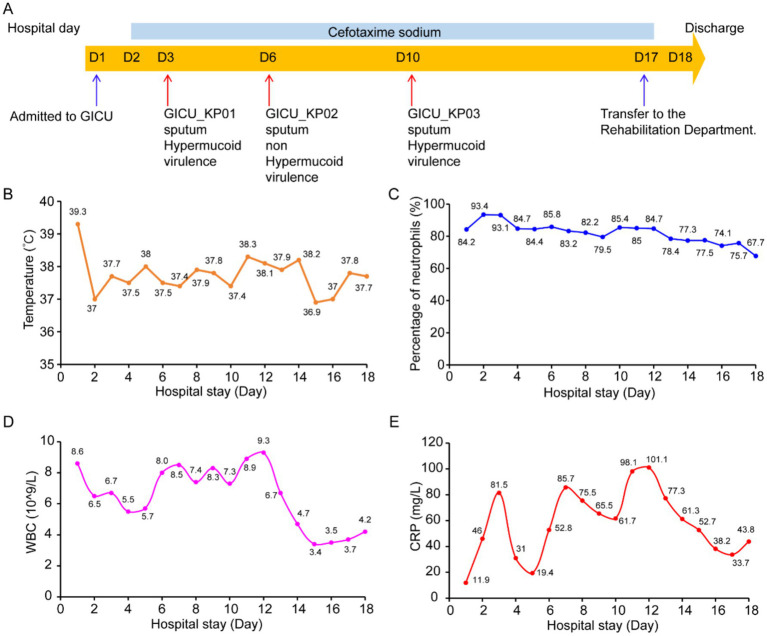
Basic characteristics of the patient. **(A)** Hospitalization history and bacterial isolation from the patient: The patient was hospitalized for 18 days and received treatment with cefotaxime (light blue bar) during this period. The blue arrows indicate the times the patient was transferred to and from the GICU, while the red arrows indicate the times *Klebsiella pneumoniae* was isolated from the patient during hospitalization and the sources of the specimens. **(B)** Dynamic changes in the patient’s temperature levels during hospitalization. **(C)** Dynamic changes in the patient’s neutrophil levels during hospitalization. **(D)** Dynamic changes in the patient’s white blood cell count during hospitalization. **(E)** Dynamic changes in the patient’s C-reactive protein levels during hospitalization.

During his GICU hospitalization, three distinct strains of *Klebsiella pneumoniae* were isolated from the patient’s sputum samples, labeled GICU_KP01, GICU_KP02, and GICU_KP03. According to the antimicrobial susceptibility results of the bacterial culture, ceftazidime was administered for infection control (Figure 1A). On the first day of admission, the patient’s highest temperature reached 39.3°C, and during the subsequent hospitalization, the patient experienced recurrent low-grade fever (Figure 1B). His white blood cell count, neutrophil percentage, and C-reactive protein (CRP) levels were continuously monitored. The data showed a consistently high neutrophil percentage (Figure 1C) and significant variations in white blood cell and CRP levels, with three notable peaks corresponding to the isolation times of the infecting *Klebsiella pneumoniae* strains (Figures 1D,E).

### Phenotypic characterization of *Klebsiella pneumoniae*

**Figure 2:**
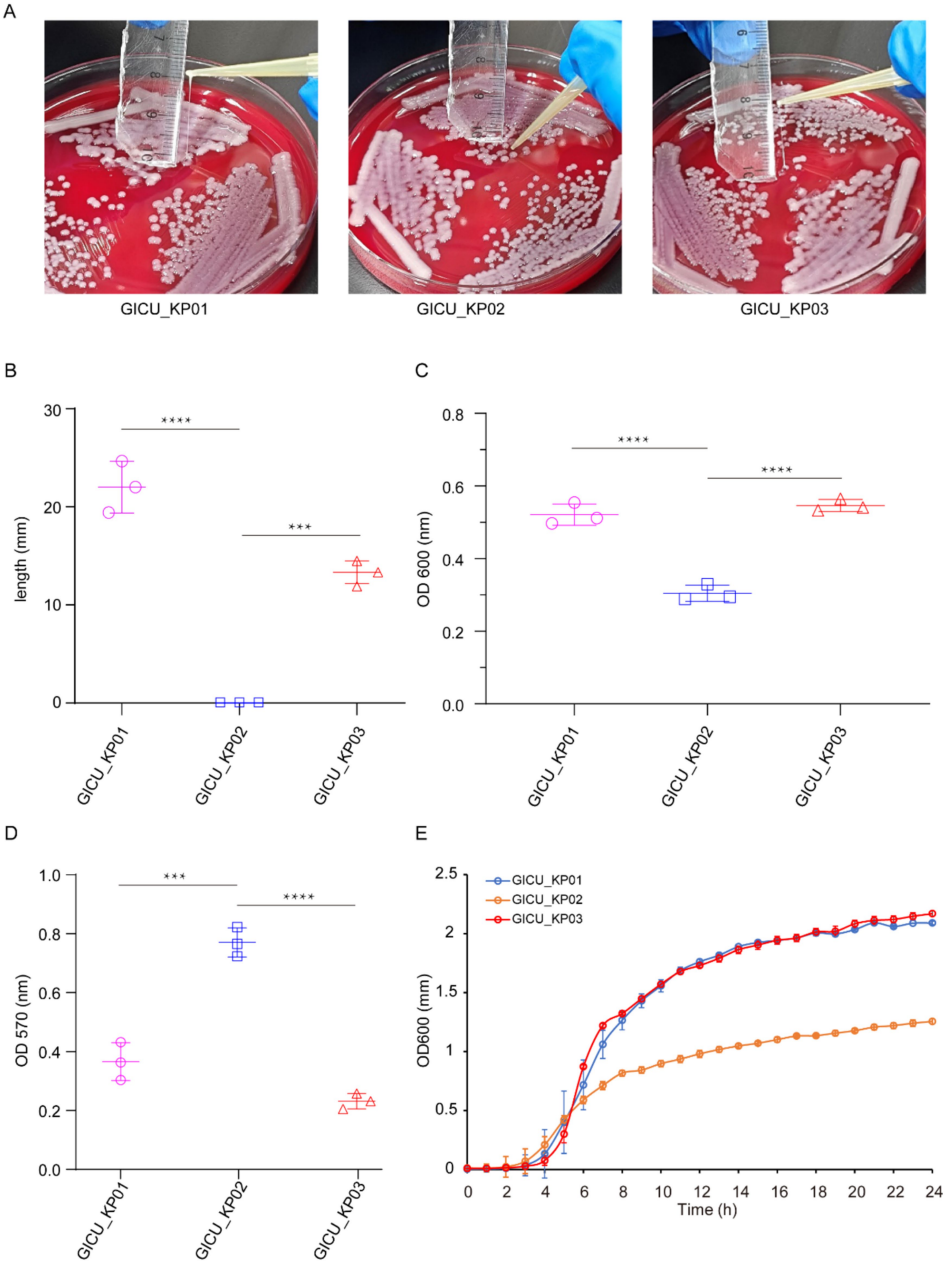
Virulence phenotypes of the three *Klebsiella pneumoniae* strains. **(A,B)** The string test on the plates determined the phenotypes of the three strains, and the lengths of the strings were measured. **(C)** Comparison of the centrifugation resistance among the three *Klebsiella pneumoniae* strains. **(D)** Comparison of biofilm thickness among the three *Klebsiella pneumoniae* strains. **(E)** Comparison of growth rates among the three *Klebsiella pneumoniae* strains. ***p < 0.001; ****p < 0.0001.

Typically, strains of the same type isolated from a patient within a short period are presumed to be identical. However, the antimicrobial susceptibility testing showed that while GICU_KP01 and GICU_KP02 had identical antibiotic sensitivities, GICU_KP03 exhibited a completely different resistance profile, including resistance to multiple antibiotics and carbapenems (Table 1). We conducted comprehensive testing on all three *Klebsiella pneumoniae* strains. Initially, bacterial morphology was assessed on blood agar using streaking techniques. The mucoid characteristic test revealed variations among the strains (Supplementary Figure S1); GICU_KP01 and GICU_KP03 tested positive in the string test, with lengths exceeding 5 mm, while GICU_KP02 tested negative, showing a gelatin-like adherence with no string formation (Figure 2A). The statistical analysis showed string lengths of approximately 22 mm for GICU_KP01, 19 mm for GICU_KP03, and 0 mm for GICU_KP02. The anti-centrifugal test demonstrated significant differences in mucoid characteristics among the strains (Figure 2B), and the mucoid viscosity sedimentation assay showed that the viscosities of GICU_KP01 and GICU_KP03 were higher than that of GICU_KP02 (Figure 2C). The quantitative biofilm assays indicated that GICU_KP01 and GICU_KP03 produced fewer biofilms than GICU_KP02 (Figure 2D). The growth curve analysis showed that GICU_KP02 had a slower growth rate and lower population in the stationary phase compared to GICU_KP01 and GICU_KP03. Moreover, during the stationary phase, the population of GICU_KP02 was lower than that of GICU_KP01 and GICU_KP03 (Figure 2E).

### Genetic analysis of the three *Klebsiella pneumoniae* strains

Based on the antimicrobial susceptibility results, we initially hypothesized that GICU_KP01 and GICU_KP02 are the same strain. However, contradictory findings from the string tests, anti-centrifugal tests, quantitative biofilm assays, and growth curve analyses suggested that GICU_KP01 and GICU_KP03 were more likely to be identical. To resolve these discrepancies and further elucidate the differences among the strains, we employed PFGE to examine the genomic and plasmid profiles of the three *Klebsiella pneumoniae* isolates. The PFGE results revealed that GICU_KP01 and GICU_KP02 displayed identical genomic banding patterns and harbored plasmids of the same size. In contrast, GICU_KP03 exhibited a unique genomic profile and carried two different plasmids (Supplementary Figure S2).

**Figure 3:**
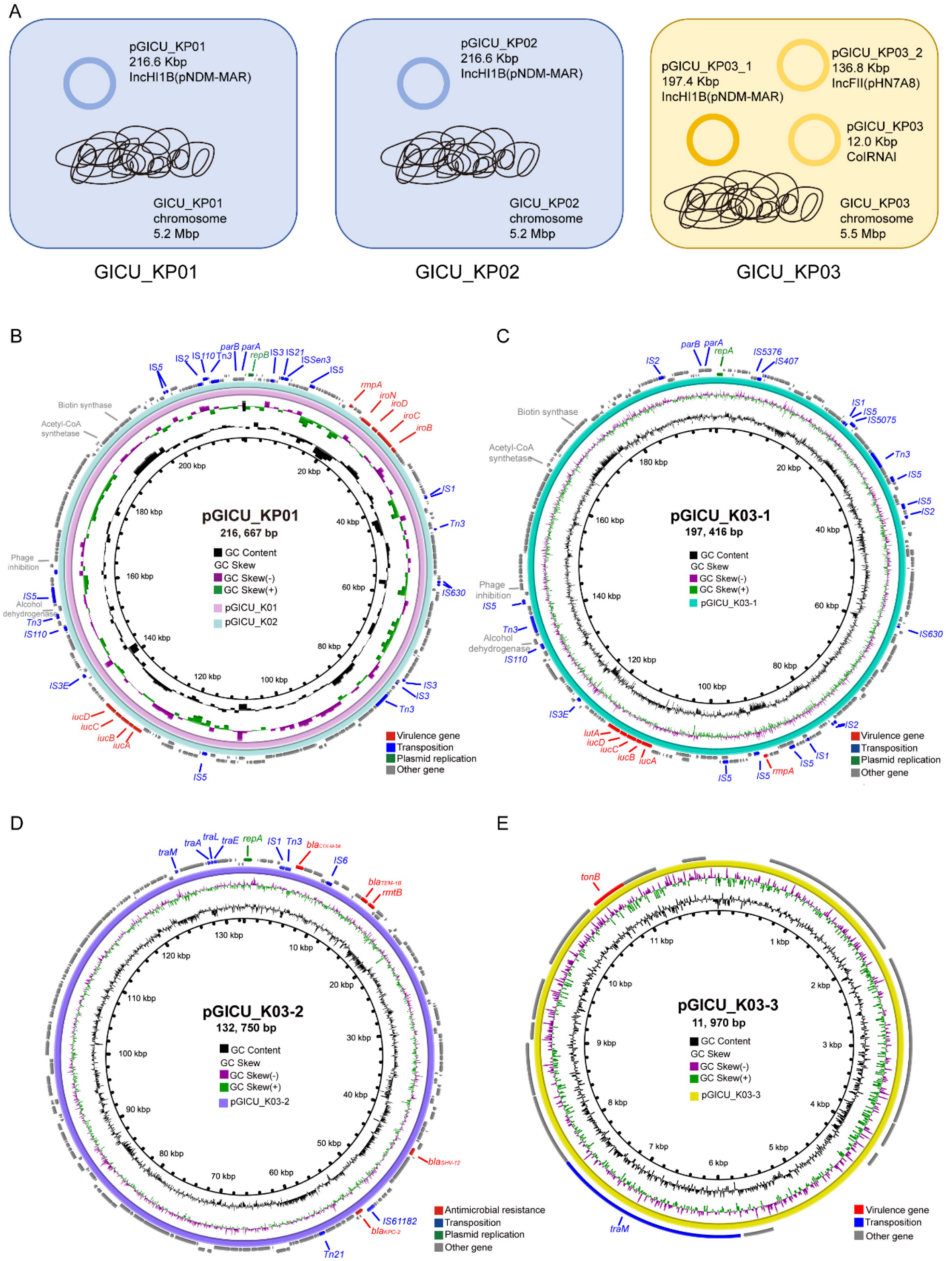
Comparative genomic analysis of the genetic backgrounds of the three strains GICU_KP01, GICU_KP02, and GICU_KP03 with different phenotypes. **(A)** The genetic backgrounds of the three *Klebsiella pneumoniae* strains, where GICU_KP01 and GICU_KP02 belonged to the ST2159-K1 type, both carrying the same plasmid and having consistent genome sizes. In contrast, the strain GICU_KP03 belonged to the ST11-K64 type and carried three different types of plasmids. **(B–E)** Comparative analysis of the plasmids carried by GICU_KP01, GICU_KP02, and GICU_KP03 using the BRIG software. The strains GICU_KP01 and GICU_KP02 carried the same plasmid, the IncHI1B plasmid, with both plasmids approximately 216.66 kb in size. GICU_KP03 carried three plasmids: (i) pGICU_KP03-1 (approximately 197.41 kb), (ii) pGICU_KP03-2 (approximately 132.75 kb), and (iii) a smaller plasmid, pGICU_KP03-3 (approximately 11.97 kb).

To gain a clearer understanding of the genetic backgrounds of these strains, we conducted WGS on all three strains. The sequencing confirmed that GICU_KP01 and GICU_KP02 are indeed the same strain, belonging to the ST2159-K59 lineage. Meanwhile, GICU_KP03 was classified under the widely distributed ST11-KL64 lineage in China (Table 2). Both GICU_KP01 and GICU_KP02 had nearly identical genomic and plasmid sizes, with plasmid types classified as virulence plasmids, consistent with the PFGE findings (Figure 3A). On the other hand, GICU_KP03 had a genome size 0.3 Mbp larger than those of the other two strains and carried three different plasmids, including two virulence plasmids and one resistance plasmid (Figure 3A).

**Table 2:** Sequence Type and K type of the three *Klebsiella pneumoniae* strains.

Strains	Lineage		Nucleotide sequence identity
	Sequence type	K-antigen serotype	
GICU_KP01	ST2159	K1	>99.99% (only 3 SNPs)
GICU_KP02	ST2159	K1	
GICU_KP03	ST11	K64	/

We conducted a comparative analysis of the virulence plasmids carried by GICU_KP01 and GICU_KP02. The plasmids pGICU_K01 and pGICU_K02 were 216,667 bp in size, with a GC content of 50%, and contained two plasmid replication initiator types—IncHI1B (pNDM-MAR) and repB (Figure 3B). pGICU_K01 and pGICU_K02 harbored multiple virulence factors, including the capsule polysaccharide regulator *rmpA*, siderophore iroBCDN, and iron-carrier-associated virulence factors *iucD, iucC, iucB,* and *iucA*. The pGICU_K03-1 plasmid carried by GICU_K03 was 197,416 base pairs in size, with a GC content of 50%. Similar to pGICU_K01 and pGICU_K02, this plasmid was also of the IncHI1B incompatibility group and harbored two plasmid replication initiator types: IncHI1B (pNDM-MAR) and *repB* (Figure 3C). It carried multiple virulence genes, including four types of siderophore clusters (*iucD, iucC, iucB, iucA*) for iron acquisition, as well as the transcriptional regulator *lutA* and the capsule polysaccharide regulator *rmpA*. The plasmid pGICU_K03-2, which was 132,750 bp in size with a GC content of 53%, contained two replication initiator types: IncR and IncFII (pHN7A8) (Figure 3D). It carried a carbapenem resistance gene *bla*_KPC-12_, three β-lactam resistance genes *bla*_CTX-M-56_, *bla*_TEM-1B_, and *bla*_SHV-12_, and an aminoglycoside resistance gene *rmtB*, but lacked any virulence factors. The plasmid pGICU_K03-3 was a ColRNAI-type plasmid of 11,970 bp with a GC content of 56% (Figure 3E). This plasmid carried only one virulence factor, *tonB*, and one transfer element, traM.

Therefore, based on the genomic sequencing results, we concluded that GICU_KP01 and GICU_KP02 belonged to the same strain, despite exhibiting distinct phenotypic differences. Consequently, we conducted a comparative genomic analysis of the two *K pneumoniae* strains and identified only three nucleotide differences between them. The first mutation was a synonymous mutation located within the glycerol dehydratase gene (Table 3). The second was a nonsense mutation situated in the promoter region of the carbohydrate porin gene (Table 3). The final mutation resulted in an amino acid change in the tyrosine protein kinase (Wzc), which is part of the capsule polysaccharide synthesis gene cluster (Table 3).

**Table 3:** Nucleotide and protein differences between GICU_KP01 and GICU_KP02.

Gene	KP01	KP02	Product	Protein change
*cpo*	C	G	Glycerol dehydratase	V216V
*pGDHL*	T	A	GDHL promoter region	/
*wzc*	A	T	tyrosine-protein kinase Wzc	N693I

### Effects of the *wzc* mutation on the mucoid properties of *Klebsiella pneumoniae*

**Figure 4:**
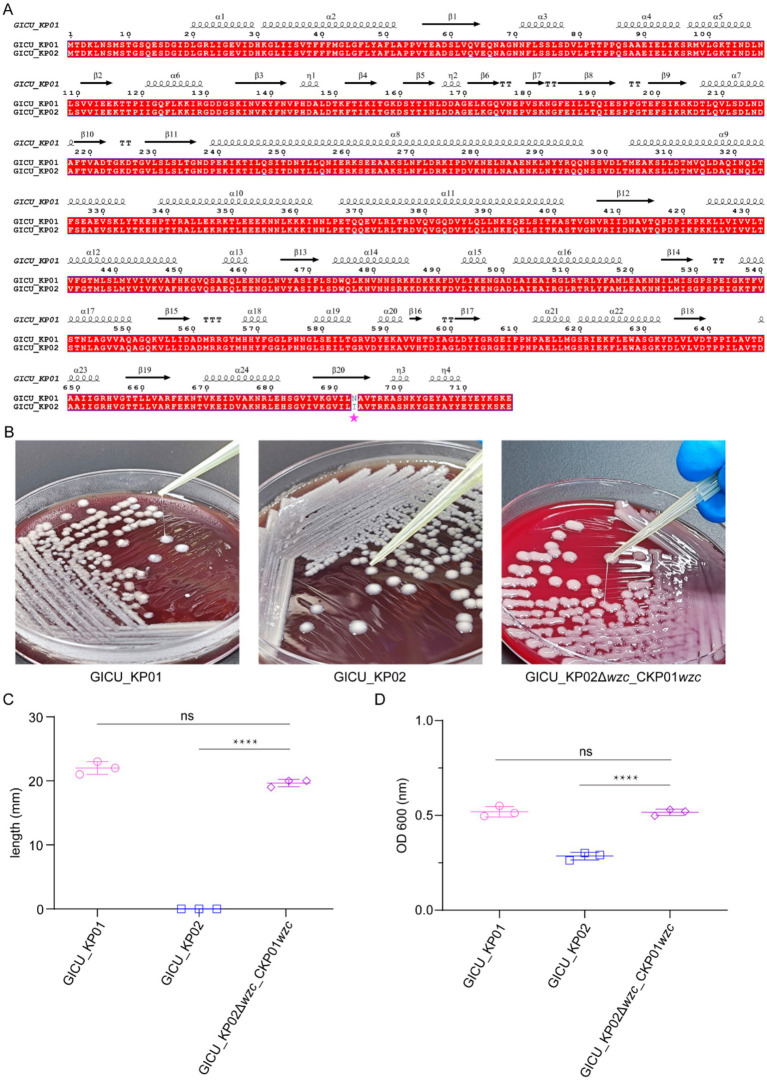
Restoration of high mucoid phenotype in GICU_KP02 by *wzc* complementation from GICU_KP01. **(A)** Alignment of the amino acid sequences of *wzc* in GICU_KP01 and GICU_KP02, with mutations indicated by asterisks. **(B,C)** Comparison of the filament length in GICU_KP01, GICU_KP02, and GICU_KP02Δ*wzc*_CKP01*wzc*, along with statistical analysis. **(D)** Statistical analysis of the centrifugation resistance levels of GICU_KP01, GICU_KP02, and GICU_KP02Δ*wzc*_CKP01*wzc*. ****p < 0.0001.

The genomic sequencing analysis suggested that a mutation in the *wzc* gene may reduce capsular polysaccharide synthesis in *Klebsiella pneumoniae*, thus altering its highly mucoid properties. Amino acid sequence alignment identified a mutation at position 693 in the bacterial capsule polysaccharide synthesis gene *wzc*, where asparagine is replaced by isoleucine (Figure 4A). To investigate this mutation’s functional impact, we employed the CRISPR/Cas9 editing system to knock out the non-functional *wzc* gene in GICU_KP02 and complement it with the functional *wzc* gene from GICU_KP01.

The string test results showed that the edited strain, GICU_KP02Δ*wzc* _CKP01 *wzc*, was positive for the string test (Figure 4B). The statistical analysis revealed that the average string length for GICU_KP02Δ*wzc* _CKP01 *wzc* was 20.3 mm, significantly different from that of GICU_KP02, yet comparable to that of GICU_KP01 (Figure 4C). Similarly, the anti-centrifugal test results indicated a significant difference in the mucoid characteristics between GICU_KP02Δ*wzc* _CKP01 *wzc* and GICU_KP02 (Supplementary Figure S3), but no significant differences were observed when compared to GICU_KP01 (Figure 4D). These results align with the string test findings, confirming the functional relevance of the *wzc* mutation in modulating the mucoid phenotype of *Klebsiella pneumoniae*.

### Assessment of *wzc* mutation impact on *Klebsiella pneumoniae* virulence using a murine sepsis model

**Figure 5:**
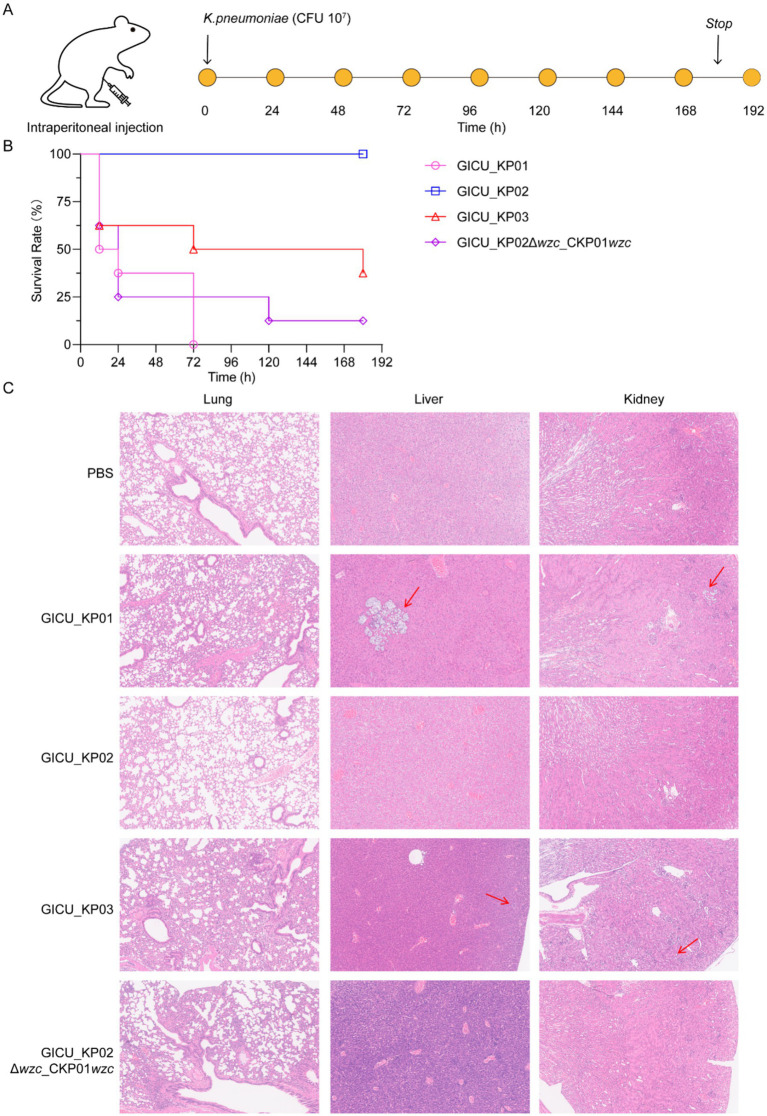
Construction of a mouse infection model to assess the virulence of the four strains of *Klebsiella pneumoniae*. **(A,B)** A mouse infection model was constructed using intraperitoneal injection, and the mice were observed continuously for 7 days to assess the virulence of the four strains of *Klebsiella pneumoniae*. **(C)** The PBS group was used as a negative control, and histopathological analysis was performed on the lungs, liver, and kidneys of the mice infected with the four strains of *Klebsiella pneumoniae*.

To definitively assess the impact of the *wzc* mutation on the virulence of *Klebsiella pneumoniae*, we established a murine sepsis model to evaluate the effects of *wzc* on bacterial virulence. CD-1 mice were inoculated intraperitoneally with various bacterial strains to test their virulence (Figure 5A). The murine sepsis model demonstrated that at an inoculum of 1 × 10^7^ CFU, both GICU_KP01 and GICU_KP03 exhibited high mortality rates, whereas GICU_KP02 was non-lethal under similar conditions post-infection for up to 180 h. Moreover, GICU_KP02Δ*wzc*_CKP01*wzc* also showed a high mortality rate, suggesting that the repair of the *wzc* mutation restores the synthesis of CPS, thereby enhancing bacterial virulence (Figure 5B). The PBS group served as a negative control, and histopathological analyses were conducted on the lungs, liver, and kidneys of the mice in the infection models of the four *Klebsiella pneumoniae* strains. The results indicated that the lung tissue pathology of the mice infected with GICU_KP01, GICU_KP03, and GICU_KP02Δ*wzc*_CKP01*wzc* showed significant exudation, while the lung tissue pathology of the mice infected with GICU_KP02 was consistent with that of the PBS group, showing no apparent pathological changes. Notably, abscess formation was observed in the liver and kidney histopathological sections of the mice infected with GICU_KP01 and GICU_KP03, with a relatively larger abscess area in the GICU_KP01 group (Figure 5C).

### *wzc* mutation impact on *Klebsiella pneumoniae* capsular synthesis

**Figure 6:**
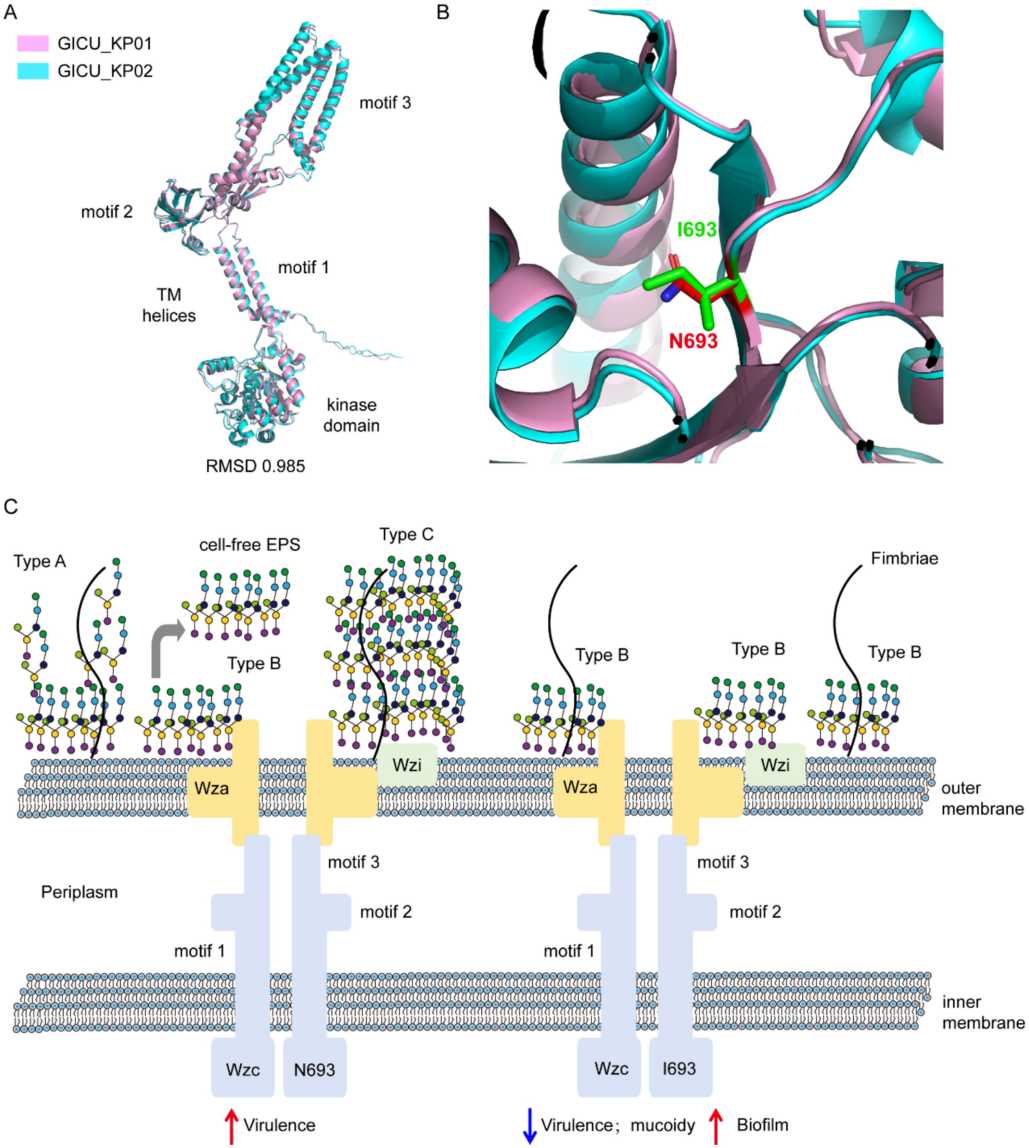
Schematic diagram illustrating the disruption of capsule synthesis in *Klebsiella pneumoniae* due to the *wzc* mutation. **(A,B)** Comparison of the structure of the *wzc* gene in the two strains of *Klebsiella pneumoniae*, GICU_KP01 and GICU_KP02. **(C)** Schematic diagram showing the reduction in bacterial capsule polysaccharide synthesis after the mutation of the two strains of *Klebsiella pneumoniae*, GICU_KP01 and GICU_KP02, leading to the exposure of fimbriae.

We utilized AlphaFold3 to predict the structures of the *wzc* proteins from GICU_KP01 and GICU_KP02, which primarily encompassed a tyrosine protein kinase domain and three modules. PyMOL calculations revealed a root mean square deviation (RMSD) value of 0.985 between the two structures, indicating that their overall *wzc* structures were largely conserved (Figure 6A). The mutation site was located within the tyrosine protein kinase domain, where asparagine at position 693 in *wzc* was mutated to isoleucine, resulting in the loss of a nitrogen atom from the side chain and a subsequent alteration in its spatial orientation (Figure 6B). This directly disrupted the synthesis of the *Klebsiella pneumoniae* capsular polysaccharide, leading to the exposure of bacterial flagella and an enhanced ability to colonize (Figure 6C).

## Discussion

Hypervirulent *Klebsiella pneumoniae* (hvKP) is a highly pathogenic bacterial strain that can cause severe infections, including pneumonia, liver abscesses, meningitis, sepsis, and others. It has the potential to cause invasive infections, even in otherwise healthy individuals. In recent years, the incidence of hvKP infections has been on the rise, garnering global attention. In 2024, the WHO updated its priority pathogen list, elevating the threat level of *Klebsiella pneumoniae* (WHO, 2024). Recent studies have shown an increase in the geographic distribution, healthcare associations, and multidrug resistance of *hypervirulent Klebsiella pneumoniae* (Liu et al., 2022). As hvKp strains evolve higher levels of antibiotic resistance and disseminate within healthcare settings, they pose a significant threat to vulnerable patient populations, potentially increasing the incidence and mortality associated with *Klebsiella pneumoniae* infections. In recent years, the emergence of hvKP strains resistant to third-generation cephalosporins, carbapenems, and polymyxins has posed significant challenges for clinical treatment and management.

The virulence determinants of hvKP include capsular polysaccharide serotypes, *rmpA* regulatory factors, iron siderophore content, virulence plasmids, type one and type three fimbriae, and the type 6 secretion system (Patel et al., 2014). hvKP exhibits a strong survival capability and resistance to neutrophil phagocytosis, which contribute to the spread and dissemination of infection. However, upon infection with these hvKP, the host immune system is also strongly activated. This leads to the production of antibodies that neutralize the toxins and other harmful substances produced by the bacteria, thereby reducing their damage to host cells. Simultaneously, the immune system identifies and eliminates infected host cells through a process known as cytotoxic T cell-mediated cellular immunity (Gao et al., 2024). In the ongoing interplay between the host and bacteria, the latter demonstrates a strong capacity for environmental adaptation through diverse genetic evolution mechanisms, allowing them to survive and proliferate in various environments and hosts (Zhang et al., 2025). Current research indicates a close relationship between bacterial adaptive evolution mechanisms and random mutations in capsular polysaccharide synthesis genes, commonly including *wzi, wza, wzb, wzc, wcaJ, rmpA,* and the *rmpA* promotor region. Among these, mutations in *wzc* and *wcaJ* are particularly prevalent, as they disrupt the normal synthesis of CPS, thereby enhancing epithelial cell invasion, biofilm formation *in vitro*, and the persistence of urinary tract infections (Ernst et al., 2020). Furthermore, the strength of *rmpA* and its promoter also influences the synthesis of CPS. Most studies have described various types of mutations in virulence genes found in the environment, which are somewhat correlated with the virulence phenotypes of *Klebsiella pneumoniae*; however, they have not provided a detailed explanation for the patterns of genetic diversity mutations observed in *Klebsiella pneumoniae*. In recent years, researchers have increasingly focused on the phenomenon of bacterial adaptive evolution within the host. For instance, Khadka et al. found that *Klebsiella pneumoniae* can counteract urine-mediated mucoid inhibition through mutations in wzc. The researchers classified the wzc mutations into three categories: those located in the periplasmic space, those in the cytoplasm, and those at the tyrosine kinase active site. These mutations have different effects on mucoidy, EPS production, and wzc autophosphorylation. Among them, mutations at the tyrosine kinase active site (G569C, G569V, G569D, and P646S) result in the loss of the hypermucoviscous phenotype (Khadka et al., 2023; Song et al., 2024). Lopatto et al. identified adaptive changes in *Klebsiella pneumoniae* during bladder colonization and successfully constructed a urinary tract infection animal model to induce targeted mutations in *Klebsiella pneumoniae* (Lopatto et al., 2024). Dai Y et al. found that missense mutations in wzc of *Klebsiella pneumoniae* are the most common mutation events. A total of 86.21% of the mutations occur within the cytoplasmic domain (amino acids (aa) 448–722), among which 51.72% are located in the ATPase active domain, especially at position 607, followed by position 647 (Dai et al., 2025). In our preliminary research, we also observed that, within the same host, *Klebsiella pneumoniae* exhibited a mutation in the *rmpA* promoter, changing from poly 11T to 10T. This resulted in reduced capsular polysaccharide synthesis, leading to decreased virulence and enhanced adaptability (Teng et al., 2024). He et al. found that some highly virulent *Klebsiella pneumoniae* strains undergo adaptive evolution in the host through mutations in *wzc* and *wzaJ*. Specifically, they discovered that mutations in *wzaJ* lead the bacteria to sacrifice some virulence in exchange for enhanced colonization ability and phagocytosis resistance. In contrast, mutations in *wzc* enable the bacteria to achieve greater adhesiveness, thereby increasing their virulence (He et al., 2023). Many isolates of *Klebsiella pneumoniae* exhibit a mucoid phenotype, characterized by the formation of moist and shiny colonies on culture media, indicating a certain degree of capsular polysaccharide production. However, these strains do not meet the “high mucoviscosity” standard in the string test (string length < 5 mm) and thus cannot be classified as having a high mucoviscosity phenotype (Shon et al., 2013). This “intermediate phenotype” is relatively common in clinical settings, suggesting that these strains exhibit some degree of mucoviscosity but do not display the typical characteristics of hvKP. Although the string test is a simple screening method that can effectively distinguish high-mucoviscosity strains, it has certain limitations in subjectivity and sensitivity when it comes to strains that fall between the typical and high-mucoviscosity phenotypes. Therefore, for strains with this intermediate phenotype, it is recommended to combine genotypic analysis (virulence genes such as *rmpA* and *magA*) with clinical manifestations to achieve a more comprehensive evaluation of their pathogenic potential (Russo et al., 2024).

We also isolated two strains of *Klebsiella pneumoniae* from the same patient, and genomic analysis indicated that the two strains were homologous. Both strains of *Klebsiella pneumoniae* simultaneously exhibited moist, viscous colonies with well-defined edges, which was somewhat misleading in determining their mucoid phenotype. We cultured the strains on both blood agar and LB agar and observed no significant morphological differences between the two strains. However, the string test results indicated that the two strains of *Klebsiella pneumoniae* exhibited different mucoid phenotypes (Singh et al., 2022; Liang et al., 2024). A mutation in the 693rd amino acid of the tyrosine kinase *wzc*, from asparagine to isoleucine, in the capsular polysaccharide synthesis gene cluster disrupted the synthesis of CPS. This directly led to a decrease in bacterial virulence while enhancing colonization ability and phagocytosis resistance. Our findings, alongside those of He et al., suggest that mutations in the tyrosine kinase *wzc* during bacterial adaptive evolution within the host exhibit randomness. However, we propose that this randomness is not solely due to random genetic mutations but may be directly linked to the host’s immune status. In immunocompetent patients, *Klebsiella pneumoniae* may reduce capsular polysaccharide synthesis to adapt to the host’s immune environment, sacrificing virulence and transmissibility in exchange for colonization and survival. Conversely, in immunocompromised patients, *Klebsiella pneumoniae* exhibits stronger virulence and transmissibility (Liu et al., 2023). This phenomenon is explained in the study by Hu et al., which found that reduced capsular polysaccharide synthesis—resulting in decreased virulence and enhanced colonization ability—may be due to mutations in capsular polysaccharide synthesis genes. These mutations prevent the bacteria from utilizing undecaprenyl phosphate (Und-P), thereby increasing its availability for the synthesis of O antigens. This results in increased biofilm formation, enhanced serum resistance, and improved evasion of antibody-mediated killing (Hu et al., 2024).

Despite identifying numerous mutation sites within the capsular polysaccharide synthesis gene cluster through environmental studies and acknowledging that *Klebsiella pneumoniae* adapts its virulence to enhance survival within the host, this study has its limitations. We isolated only a single strain from one patient, which limits our ability to determine whether the adaptive changes observed in the bacteria within the host are inevitable phenomena or merely random occurrences. Serving as a starting point, this study will be followed by our continuous efforts to obtain more bacterial strains and attempt to elucidate the patterns of adaptive evolution in the interactions between bacteria and their hosts.

## Conclusion

We investigated the *in vivo* evolution of two ST2159-K1 strains that shared similar genotypes but exhibited different phenotypic characteristics. Our findings suggest that *Klebsiella pneumoniae* evolution occurs not only in the environment but also within the human body. *Klebsiella pneumoniae* may undergo different adaptive changes based on varying host responses, typically achieved through the enhancement or attenuation of capsular polysaccharide synthesis to adapt to environmental conditions.

## Data Availability

The datasets presented in this study can be found in online repositories. The names of the repository/repositories and accession number(s) can be found below: https://www.ncbi.nlm.nih.gov/genbank/, PRJNA1138907; https://www.ncbi.nlm.nih.gov/genbank/, PRJNA1071829.

## Footnotes

^1^ https://rast.nmpdr.org

^2^ https://cge.food.dtu.dk/services/PlasmidFinder

^3^ https://cge.food.dtu.dk/services/ResFinder

^4^ https://www-is.biotoul.fr/index.php

^5^ https://bioinfo-mml.sjtu.edu.cn/oriTfinder

^6^ https://golgi.sandbox.google.com/
