# Supplementary material for: The wzc mutation mediates virulence changes in K1-type Klebsiella pneumoniae within the same patient
Source: Front Microbiol. 2025 May 15;16:1577629. doi: 10.3389/fmicb.2025.1577629 (PMC12119577; doi:10.3389/fmicb.2025.1577629)
Supplement: Supplementary file 1 [file Data_Sheet_1.doc]

**Supplemental Figures**

**Figure S1: Klebsiella pneumoniae morphology and size.**


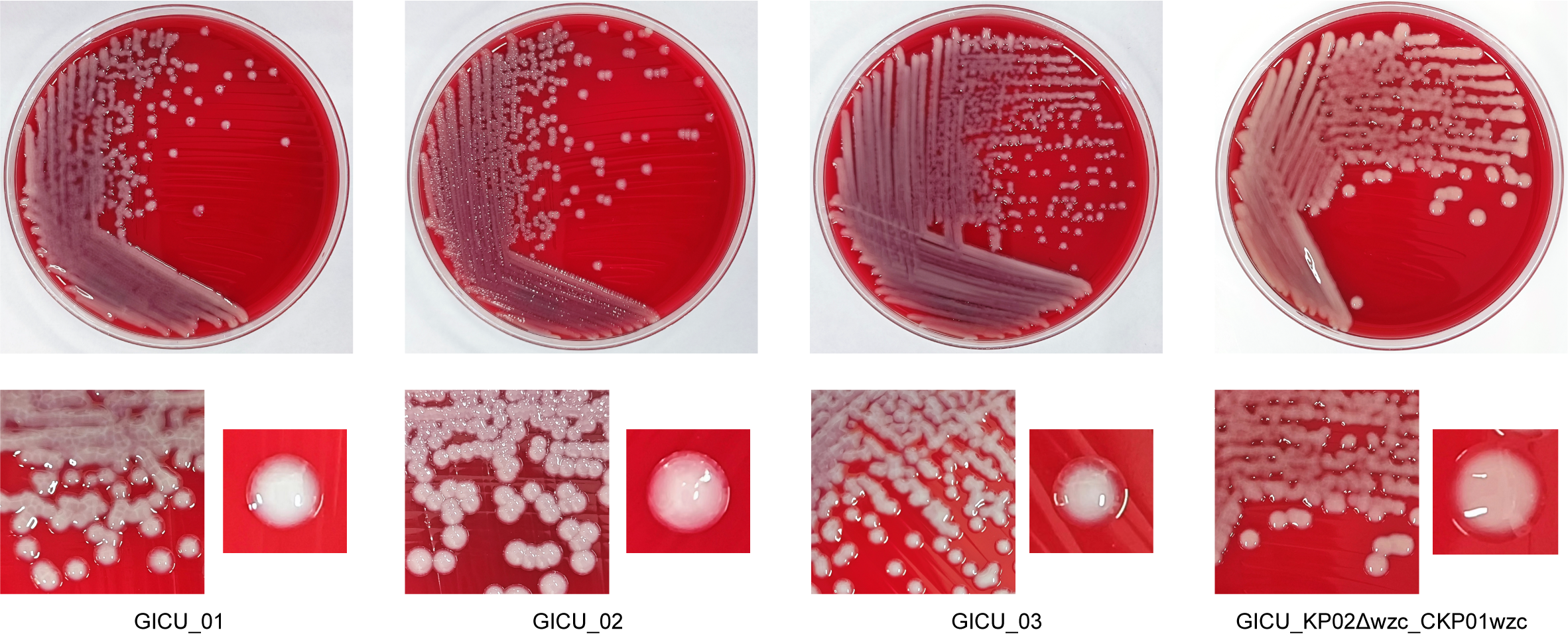


**Figure S2: Klebsiella pneumoniae Pulsed-field Gel Electrophoresis.**


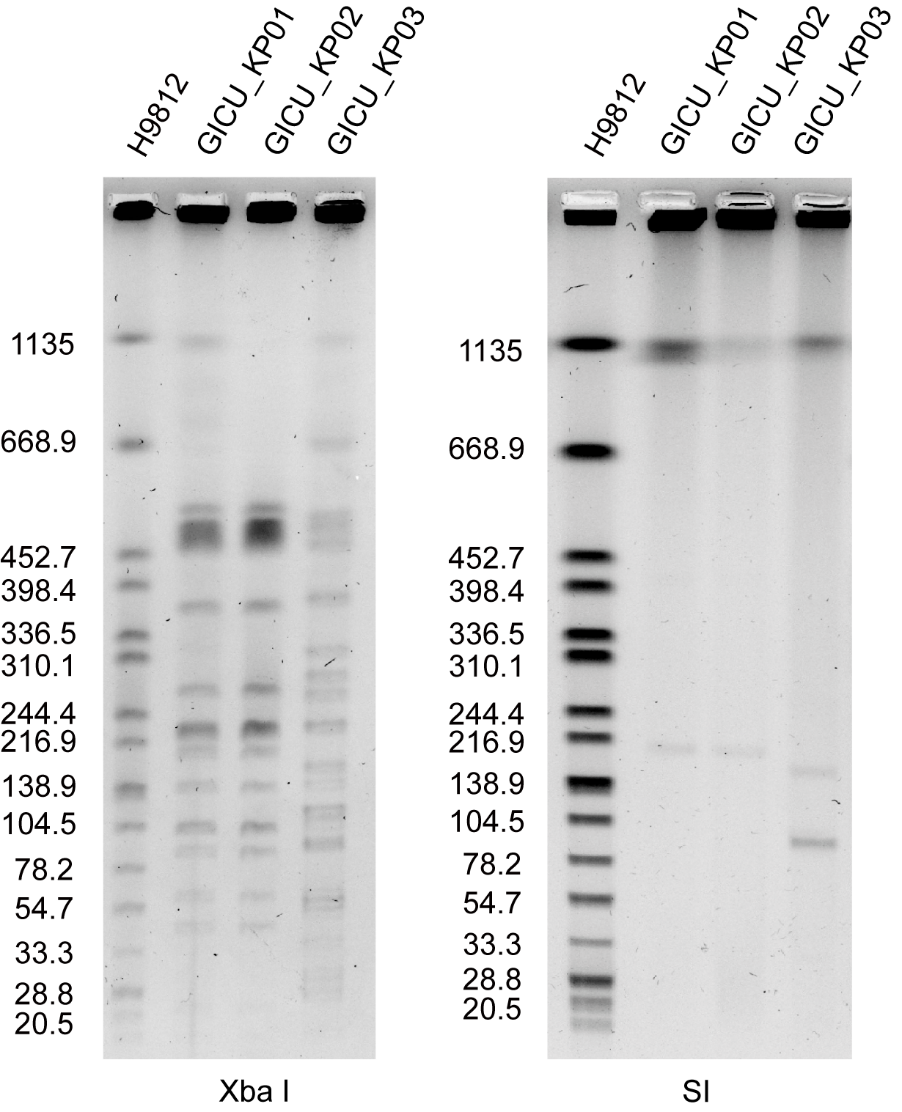


**Figure S3: Klebsiella pneumoniae mucoid characteristics.**

**
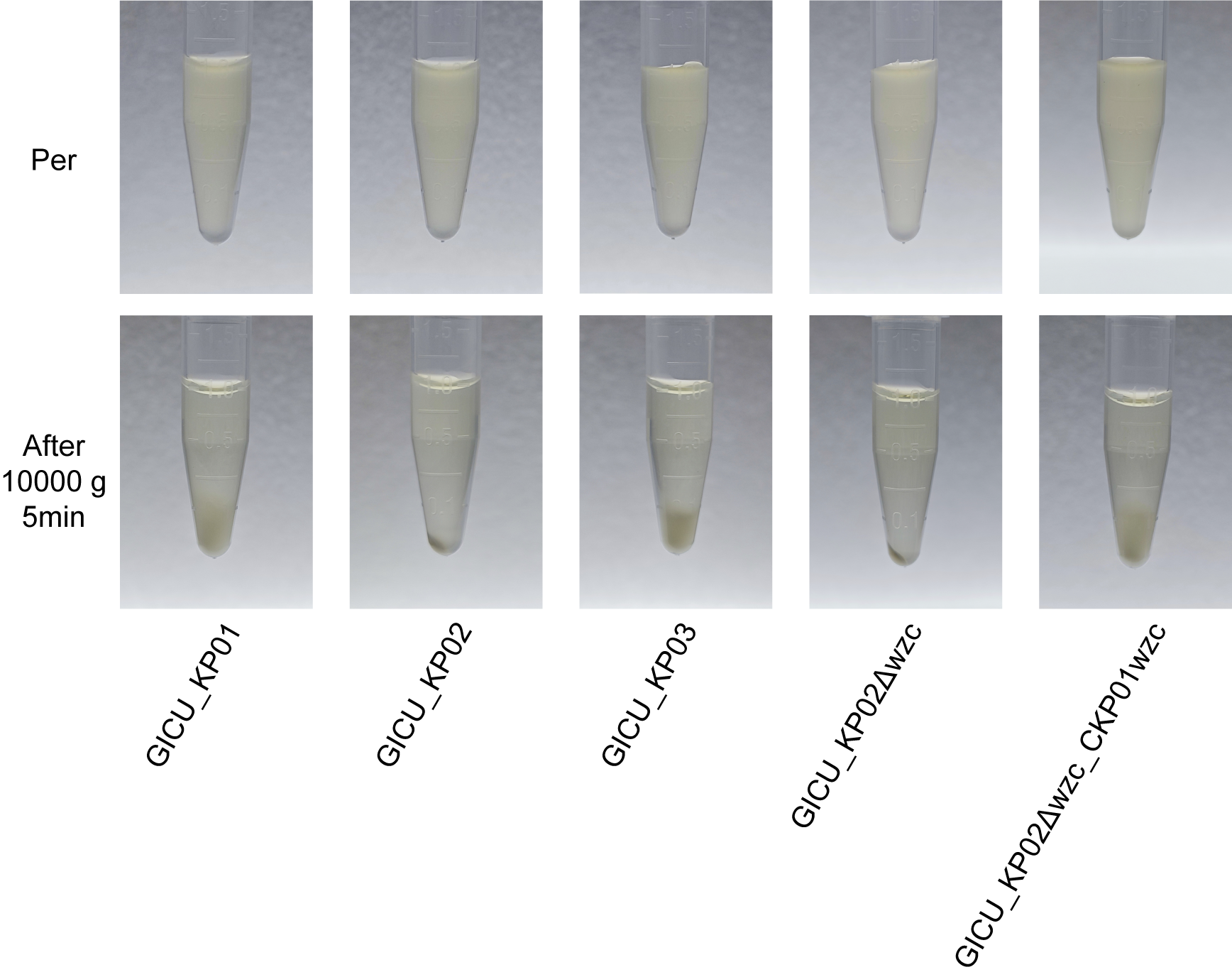
**
