# Supplementary material for: The wzc mutation mediates virulence changes in K1-type Klebsiella pneumoniae within the same patient
Source: Front Microbiol. 2025 May 15;16:1577629. doi: 10.3389/fmicb.2025.1577629 (PMC12119577; doi:10.3389/fmicb.2025.1577629)
Supplement: Supplementary file 2 [file Data_Sheet_2.docx]

**Supporting tables**

**Table S1** Bacterial strains and plasmids used in this study

| **Strains** | **Description** | **Origins** |
| --- | --- | --- |
| DH5α | A cloning host of *E. coli* | Lab stock |
| GICU_KP01 | A strain isolated from clinical | This work |
| GICU_KP02 | A strain isolated from clinical | This work |
| GICU_KP03 | A strain isolated from clinical | This work |
| KP02Δ*wzc* | KP02 that knocked out *wzc* | This work |
| KP02Δ*wzc*::KP01 *wzc* | KP02 that knocked out *wzc* then complemented KP01 *wzc* | This work |
| DH5α pSGKP::spacer | DH5α carried pSGKP*::wzc* spacer | This work |
| DH5α pSGKP*::*spacer UD | DH5α carried pSGKP*::wzc* spacer UD | This work |
| DH5α pSGKP*::*KP01 *wzc* | DH5α carried pSGKP::KP01 *wzc* | This work |
| **Plasmids** | **Description** | **Origins** |
| pSGKP | a CRISPR edit and expression vector, Rif^R^ | Lab stock |
| pCasKP | a CRISPR edit vector with Caskp, Apr^R^ | Lab stock |
| pSGKP*::wzc* spacer | pSGKP with *wzc* spacer*,* Rif^R^ | This work |
| pSGKP*::wzc* spacer UD | pSGKP with *wzc* spacer and donor DNA*,* Rif^R^ | This work |
| pSGKP*::*KP01 wzc | pSGKP with KP01 *wzc,* Rif^R^ | This work |

**Table S2** Primers used in this study

| **Primers** | **Sequences** |
| --- | --- |
| *wzc* spacer-F | 5'-GTC CTA GGT ATA ATA CTA GTC AGT TTT TAA AGA AAA TAC GGT TTT AGA GCT AGA AAT AG-3' |
| *wzc* spacer-R | 5'-CTA TTT CTA GCT CTA AAA CCG TAT TTT CTT TAA AAA CTG ACT AGT ATT ATA CCT AGG AC-3' |
| *wzc*-spacer-id | 5'-CAG TTT TTA AAG AAA ATA CG-3' |
| M13R | 5'-CAG GAA ACA GCT ATG ACC-3' |
| pSGKP-*wzc*-up-F | 5'-GAA TTC CTG CAG CCC GGG GGA TCC GAG ATA TGT AAG GAA CTG G-3' |
| pSGKP-*wzc*-up-R | 5'-GGG AAA TTA ATT TAG CTT ATC CTA GAT TTA ATT AGT TCA A-3' |
| pSGKP-*wzc*-do-F | 5'-TTG AAC TAA TTA AAT CTA GGA TAA GCT AAA TTA ATT TCC C-3' |
| pSGKP-*wzc*-do-R | 5'-CGC GGT GGC GGC CGC TCT ATG ACT GAA AGG TAT TCC TGC-3' |
| *wzc*-out-F | 5'-GAT GCA TCT GCC ATG GTA CT-3' |
| *wzc*-out-R | 5'-CGA AAG TTC AGC CCA GAT AC-3' |
| pSGKP-KP01*wzc*-F | 5'- GAA TTC CTG CAG CCC GGG GGA TCC CTA GCT CCG GAA TTA CGT GG-3' |
| pSGKP-KP01*wzc*-R | 5'- CGC GGT GGC GGC CGC TCT AGA GCA CGT CCA AGC GAG CAT AA-3' |
|  |  |
